# Nuclear–Electronic Orbital Theory: Exploring Nuclear Quantum Effects in Chemistry, Biology, and Materials

**DOI:** 10.1021/jacs.6c15297

**Published:** 2026-10-05

**Authors:** Sharon Hammes-Schiffer

**Affiliations:** Department of Chemistry, 6740 Princeton University, Princeton, New Jersey 08544, United States

## Abstract

Nuclear quantum effects play a critical role throughout chemistry, biology, and materials science. This Perspective presents the nuclear–electronic orbital (NEO) framework as a viable approach for incorporating nuclear quantum effects into calculations of relatively large, complex systems. The NEO approach treats specified nuclei, often protons or deuterons, quantum mechanically on the same level as the electrons using wave function theories or density functional theory. This approach incorporates nuclear quantum effects such as zero-point energy, nuclear delocalization, and nuclear tunneling into quantum chemistry calculations and molecular dynamics simulations. Nonadiabatic effects beyond the standard Born–Oppenheimer approximation between electrons and nuclei are also incorporated. NEO methods are computationally practical, straightforward to use, and accessible to nonexperts. This Perspective highlights the broad applicability of the NEO framework and emphasizes the fundamental insights provided by NEO calculations. The applications discussed include water clusters, double proton transfer dynamics, proton-coupled electron transfer, hydrogen tunneling dynamics, enzymatic reactions, p*K*_a_ calculations, kinetic isotope effects, hydrogen and deuterium adsorption and water monolayers at metal electrode surfaces, geometric isotope effects, phase transitions in hydrogen-rich materials such as superconducting hydrides and ice, photoinduced proton transfer, plasmon-induced H_2_ dissociation, vibrational spectroscopy, and polaritonic chemistry. These examples showcase the ubiquitous importance of nuclear quantum effects as well as the accessibility of the NEO approach.

## Graphical abstract

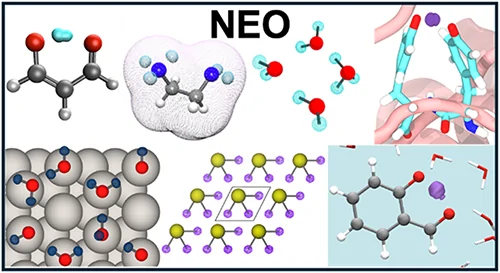

## Introduction

Although sometimes overlooked, nuclear quantum effects play a critical role in many chemical and biological processes. Nuclear quantum effects are responsible for zero-point energy, nuclear tunneling, and vibrationally excited states. Thus, they impact chemical equilibria, reaction rates, and mechanisms as well as photochemistry. Because of its light mass, the quantum nature of hydrogen is especially significant. Hydrogen nuclear quantum effects strongly impact hydrogen-bonding interactions and hydrogen transfer reactions, including proton, hydride, hydrogen atom, and proton-coupled electron transfer reactions. The fundamental roles of zero-point energy, nuclear delocalization, and hydrogen tunneling in such processes have been shown through a wide range of theoretical studies.^1–8^

Experimental evidence of the significance of nuclear quantum effects in chemistry is also available. A compelling example is proton-coupled electron transfer (PCET) in soybean lipoxygenase, which has been shown to have a kinetic isotope effect of around 80 at room temperature for the wild-type enzyme and around 700 for a double mutant.^9–11^ In other words, replacing the transferring hydrogen with deuterium decreases the rate constant by a factor of 80 or 700, respectively. Such large kinetic isotope effects are hallmarks of hydrogen tunneling. Another example can be found in studies of hydrogen-rich materials such as superconducting hydrides and ice. In these materials, the experimentally measured phase-transition pressure cannot be reproduced without treating the hydrogen nuclei quantum mechanically because nuclear delocalization plays an essential role at high pressures.^12,13^ In addition to these two particularly illustrative examples, most chemical processes involve some degree of quantum behavior associated with nuclei.

Despite the importance of nuclear quantum effects, chemists performing standard quantum chemistry calculations often either neglect them entirely or treat them at a low level by including zero-point energy based on the harmonic approximation. To address this gap, my group has been developing the nuclear–electronic orbital (NEO) framework,^14–16^ which enables the straightforward inclusion of nuclear quantum effects in quantum chemistry calculations and molecular dynamics simulations. Our goal has been to provide an approach that is efficient, accurate, accessible, and applicable to large chemical, biological, and materials systems. In the NEO framework, specified nuclei, often protons or deuterons, are treated quantum mechanically on the same level as the electrons. In this manner, nuclear delocalization, anharmonic zero-point energy, and hydrogen tunneling are incorporated into energy calculations, geometry optimizations, reaction paths, and dynamics. The NEO framework also extends beyond the common Born–Oppenheimer approximation, which assumes that electrons respond instantaneously to nuclear motion. Specifically, the Born–Oppenheimer separation is not invoked between the electrons and quantum nuclei.

A schematic comparison of conventional Born–Oppenheimer methods and NEO methods is shown in Figure 1. For simplicity, here we will assume that the quantum nuclei are protons, and we will call the nuclei that are not treated quantum mechanically within the NEO framework as “classical”. In conventional Born–Oppenheimer methods, the electrons are treated quantum mechanically, and the nuclei are treated as classical point charges. For each nuclear configuration, the electronic Schrödinger equation is solved to obtain the electronic energy, and repeating this procedure for different nuclear configurations maps out an electronic potential energy surface. To simulate dynamics, typically the nuclei move classically on this surface, although they can also move via quantum dynamics or nonadiabatic dynamics over multiple electronic surfaces.

**Figure 1:**
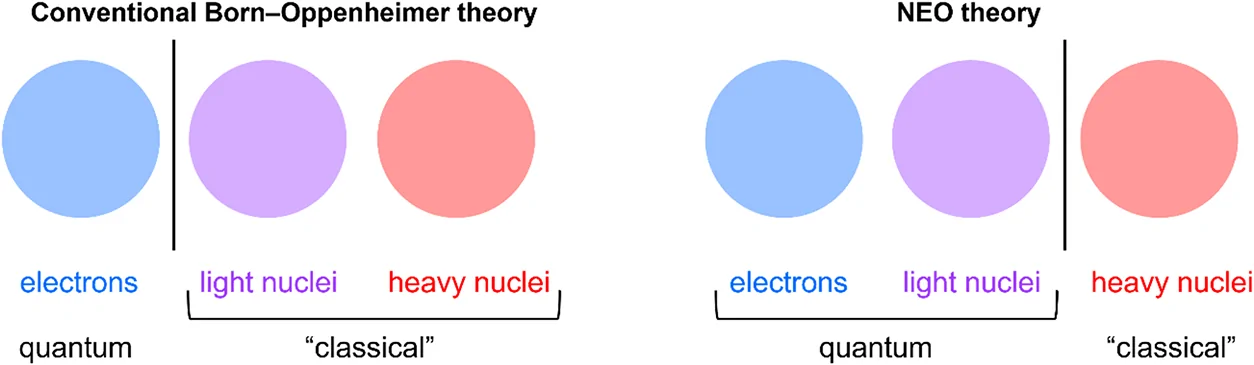
Comparison of conventional Born–Oppenheimer theory and NEO theory. In conventional methods, the electrons are treated quantum mechanically, and all nuclei are treated as classical point charges. In NEO methods, specified light nuclei such as protons are treated quantum mechanically on the same level as the electrons with no Born–Oppenheimer separation between them.

In contrast, the NEO approach treats electrons and specified nuclei quantum mechanically on the same level with no Born–Oppenheimer separation between them. For each classical nuclear configuration, a mixed nuclear–electronic Schrödinger equation is solved to obtain the vibronic energy, and repeating this procedure for different classical nuclear configurations maps out a vibronic energy surface. Analogous to conventional Born–Oppenheimer methods, the “classical” nuclei can move classically or quantum dynamically on this surface or on multiple vibronic surfaces. We emphasize that many other theoretical methods have been developed to incorporate nuclear quantum effects and non-Born–Oppenheimer effects into simulations of chemical systems.^17–29^ What sets the NEO approach apart from most of these other methods is that it is accessible to nonexperts and is applicable to large, complex chemical, biological, and materials systems. NEO calculations are nearly as straightforward to perform as are their conventional electronic counterparts.

In this Perspective, we summarize various NEO methods and provide examples of how this approach can provide experimentally relevant insights and predictions. For each method, we present at least one application to chemistry, biology, or materials science to showcase the capabilities of the NEO approach in diverse areas. The applications will include protonated water clusters, proton-coupled electron transfer in the enzyme ribonucleotide reductase, hydrogen and deuterium adsorption and water monolayers on Pt(111) surfaces, high-pressure superconducting hydrides, the ice VIII to ice X phase transition, vibrational spectroscopy, and p*K*_a_ and kinetic isotope effect calculations. Additional applications will highlight the real-time dynamics of ground-state and photoinduced proton transfer reactions, hydrogen tunneling splittings and dynamics, and plasmonic and polaritonic chemistry. Due to space limitations, the full background and details of each method and each application will not be presented, but rather, the relevant references will be cited. By clarifying the capabilities and current limitations of the NEO approach, we aim to stimulate new research directions in this field and encourage the chemistry community to explore a wide range of applications.

## NEO Wave Function Methods

In the NEO framework, the system is divided into electrons, quantum nuclei, and other nuclei that are denoted “classical”. For convenience, here we will assume that the quantum nuclei are protons, although the method can be extended to other quantum nuclei or other types of particles such as positrons.^30–33^ The NEO Hamiltonian includes the kinetic energies of the electrons and quantum protons, as well as the Coulomb interactions among the electrons, quantum protons, and classical nuclei. Although in its original conception the NEO method required at least two classical nuclei,^14^ current implementations of the real-time NEO methods^34^ as well as constrained NEO molecular dynamics methods^35^ allow all nuclei to be quantum mechanical. However, the true power of the NEO method is its applications to larger systems that would be challenging if all nuclei were treated quantum mechanically. A variety of multicomponent wave function and density functional theory (DFT) methods have been investigated by many groups over the years.^36–46^ Within this context, the goal of the NEO framework is to treat specified nuclei quantum mechanically in a manner that is accurate according to benchmarking analyses, accessible to nonexperts, and broadly applicable to complex systems. We have implemented or participated in the implementation of NEO methods into Q-Chem,^47^ Chronus Quantum,^48^ and FHI-aims.^49^ Moreover, NEO methods are also being implemented in additional software packages by other groups.

### NEO Hartree–Fock

The simplest NEO theory is based on the NEO Hartree–Fock (NEO-HF) wave function^14^

1 $$\Psi_{\mathrm{NEO}‐\mathrm{HF}}\left(x^{e},x^{p}\right)=\Phi^{e}\left(x^{e}\right)\Phi^{p}\left(x^{p}\right)$$

where **x** ^e/p^ are the collective spatial and spin coordinates of the electrons/protons. The electronic and protonic Slater determinants, Φ^e^(**x** ^e^) and Φ^p^(**x** ^p^), are composed of electronic and nuclear orbitals, respectively. Analogous to the electronic orbitals, the nuclear orbitals can be expanded in Gaussian basis sets. Given the larger mass of a proton compared to an electron, the protonic basis functions are much more localized than their electronic counterparts.^50^ Nevertheless, the mathematical treatment is identical, and the existing integral codes in electronic structure packages can be utilized. The variational method can be used to minimize the energy with respect to the electronic and nuclear orbital coefficients, leading to the electronic and protonic Hartree–Fock–Roothaan equations

2 $$\begin{array}{l} F^{e}C^{e}=S^{e}C^{e}\varepsilon^{e}\\ F^{p}C^{p}=S^{p}C^{p}\varepsilon^{p} \end{array}$$

Here, **F** ^e/p^, **C** ^e/p^, **S** ^e/p^, and **ε** ^e/p^ are the electronic/protonic Fock matrix, orbital coefficient matrix, overlap matrix, and orbital energy matrix, respectively. The form of these equations is the same as the analogous equations in conventional electronic Hartree–Fock theory. However, these two sets of electronic and protonic equations are strongly coupled to each other and must be solved self-consistently. In addition, the position of the protonic basis function centers can also be optimized variationally in conjunction with this self-consistent field procedure.

Although this approach is straightforward, it is missing electron–proton correlation, which has been shown to be critical for obtaining even qualitatively reasonable zero-point energies, proton densities, and associated physical properties.^14,44,51^ In conventional electronic structure theory, a wide range of methods have been developed to describe electron–electron correlation. However, electron–proton correlation is even more important because an electron and a proton have opposite charge and therefore attract each other, leading to especially significant correlation. To address this challenge, we have expended substantial effort developing methods that can include both electron–electron and electron–proton correlation in a computationally tractable manner. A variety of wave function methods have been explored, including perturbation theory,^52,53^ coupled-cluster theory,^54–58^ and orbital-optimized second-order perturbation theory^59,60^ for ground states and equation-of-motion^61^ and multireference methods^14,62–64^ for excited states. We have also explored multicomponent DFT,^38,65–69^ which extends conventional electronic DFT to systems with multiple types of quantum particles. An advantage of wave function methods is that they are systematically improvable and typically parameter-free. However, DFT tends to be more computationally practical for larger systems and thus is often the workhorse for large-scale applications. The remainder of this section will focus on NEO wave function methods, and the next section will discuss NEO-DFT.

In addition to the Slater determinant representation of the protonic wave function given in eq ([Disp-formula eq1]), we have also implemented NEO methods with the Hartree product representation of the protonic wave function.^70,71^ Both representations are applicable to Fermionic nuclei, such as protons, and Bosonic nuclei, such as deuterons, because the exchange terms are negligible for molecular systems with relatively localized nuclear orbitals. In particular, the exchange terms between nuclei such as protons or deuterons have been shown to be around 8–10 orders of magnitude smaller than their electronic counterparts.^44^ Moreover, the single occupation of each nuclear orbital is a valid assumption based on energetic considerations. In many cases, the nuclear Hartree product representation is more computationally efficient^71^ and improves the accuracy of perturbative corrections, most likely because of more physically reasonable virtual orbitals. Additionally, the formalisms described herein can easily be extended to systems with more than two types of quantum particles, such as electrons, protons, and deuterons or electrons, protons, and positrons. In these extensions, each type of quantum particle can be represented by either a Slater determinant or a Hartree product.

### NEO Coupled-Cluster Methods

NEO coupled-cluster (NEO-CC) methods are analogous to their electronic counterparts^72^ except that more types of excitations are included. The NEO-CC wave function can be expressed as Ψ_NEO‑CC_ = *e^T̂^* Ψ_NEO‑HF_, where the cluster excitation operator *T̂* generates excited determinants by acting on the NEO-HF wave function.^54^ For NEO-CCSD, single electron, single proton, double electron, double proton, and double electron–proton excitations are included. We have also implemented full electron–electron–proton and electron–proton–proton triple excitations, as well as perturbative treatments of these triple excitations and triple electron excitations, denoted NEO-CCSD­(T).^58^ Given that the perturbative treatments of triple excitations agree well with the full treatments, we consider NEO-CCSD­(T) to be the “gold standard” for ground-state calculations within the NEO framework. We have also developed approximate methods such as orbital-optimized second-order perturbation theory (NEO-OOMP2) and scaled variants,^59,60,73,74^ which are more computationally tractable and, in some cases, achieve similar levels of accuracy. NEO second-order coupled-cluster methods are also an efficient option for both ground and excited states.^33,56^

An application that highlights these methods is the energy ordering of protonated water tetramers. The electronic energies computed with CCSD­(T) for the four isomers shown in Figure 2 increase from left to right.^75^ Including zero-point energy (ZPE) changes this ordering such that the *cis*-Zundel and *trans*-Zundel isomers are lower in energy than the ring isomer. However, computing the ZPE is expensive at the CCSD­(T) level because it requires the calculation of a nuclear coordinate Hessian (i.e., second-derivative matrix) for a normal mode analysis as well as additional calculations to include anharmonicity. Instead, we performed NEO-CCSD and NEO-CCSD­(T) single-point energy calculations with all nine protons treated quantum mechanically.^55,58^ These NEO methods predict accurate relative energies without requiring an expensive Hessian calculation or anharmonic corrections. Moreover, the scaled variant of NEO-OOMP2 also provides accurate relative energies at a lower computational cost.^60^

**Figure 2:**
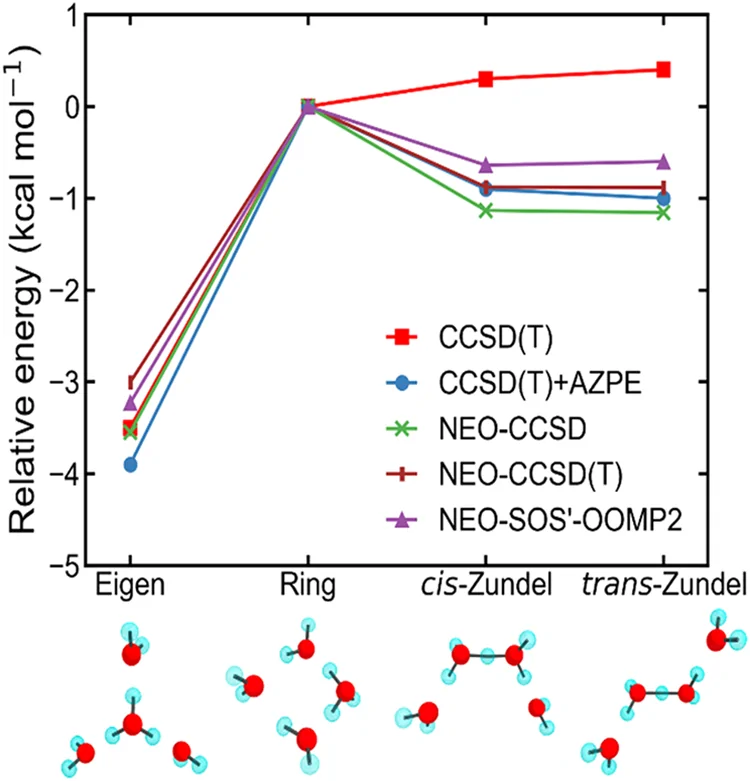
Relative energies of protonated water tetramer isomers computed with different computational methods for geometries optimized with conventional electronic CCSD­(T). The anharmonic zero-point energy (AZPE) added to CCSD­(T) was computed by adding the anharmonic ZPE at the MP2 level to the harmonic ZPE at the CCSD­(T) level and subtracting the harmonic ZPE at the MP2 level. Neglecting the anharmonic effects impacts the relative energies. All three NEO methods include the anharmonic ZPE associated with the protons from a single-point energy calculation. The ZPE associated with the oxygen atoms does not significantly impact the relative energies. Reprinted from ref 58 with the permission of AIP Publishing.

In contrast to the inclusion of ZPE through a normal mode analysis, a NEO single-point energy calculation inherently includes the anharmonicity associated with the quantum protons. We have found that including anharmonic ZPE as opposed to only harmonic ZPE can change the ordering of protonated water hexamer isomers.^60^ As will be shown below, anharmonic effects can also be important for computing accurate p*K*_a_ values and kinetic isotope effects. NEO-CCSD­(T) is the current gold standard for computing anharmonic ZPE within the NEO framework, and in some cases, it can be used to apply energy corrections to lower-level NEO calculations. With recent technical advances in NEO coupled-cluster theory, we have now performed NEO-CCSD­(T) calculations on larger water clusters with up to 24 quantum protons using reasonable local computational resources. Even larger systems will become accessible when embedding or localized orbital methods are applied to NEO-CCSD­(T).

### NEO Multiconfigurational Methods

To describe excited vibronic states and multireference problems, we have developed NEO configuration interaction (NEO-CI), complete active space self-consistent field (NEO-CASSCF),^14^ and multireference configuration interaction (NEO-MRCI)^64^ methods. These methods are all natural extensions of their conventional electronic structure theory counterparts. The NEO wave function is expressed as a linear combination of products of electronic and nuclear Slater determinants

3 $$\Psi_{I}\left(x^{e},x^{p}\right)=\sum_{i,j}C_{\mathrm{ij}}^{I}\Phi_{i}^{e}\left(x^{e}\right)\Phi_{j}^{p}\left(x^{p}\right)$$

In NEO-CI, the energy is minimized by varying the expansion coefficients, whereas in NEO-CASSCF, the electronic and nuclear orbital coefficients are also varied. In the latter case, both electronic and nuclear active spaces must be defined, and state-averaging can be performed over a range of vibronic states. Building upon the static correlation provided by NEO-CASSCF, NEO-MRCI includes excitations from multiple references in a manner that also includes dynamic correlation.^64^ With a modification of the conventional electronic basis functions to account for delocalized protons,^64^ NEO-MRCI has been shown to produce accurate vibrational excitations for small molecular systems when compared to a numerically exact grid-based method.^76^

NEO-MRCI has also been shown to produce accurate tunneling splittings for molecules at fixed geometries.^77^ Hydrogen tunneling has typically required multireference treatments within the NEO framework to achieve the bilobal proton densities characteristic of tunneling in symmetric double-well potentials (see proton densities for malonaldehyde in Figure 3). To describe such systems, we use two protonic basis function centers to represent the tunneling hydrogen, with both electronic and protonic basis functions at each center. The tunneling splitting is defined as the energy difference between the lowest two vibronic states, which correspond to the symmetric and antisymmetric bilobal protonic wave functions. The accuracy of the tunneling splittings for linear molecules at a range of donor–acceptor distances and for malonaldehyde at two different donor–acceptor distances has been verified by comparison to numerically exact grid-based methods (Figure 3).^77^ Note that many of these tunneling splittings are an order of magnitude or more below 1 kcal/mol (i.e., 1 cm^–1^ is around 0.003 kcal/mol). Thus, these benchmarking results represent a stringent test for the NEO-MRCI method, and errors on the order of 20% for the smaller splittings are considered acceptable in the context of quantum chemistry calculations. Note also that tunneling splittings computed at a single, fixed geometry are not expected to agree with experimental measurements. Experimentally relevant calculations will go beyond this static picture by including the motion of the other nuclei and their coupling to the hydrogen tunneling process. The development of such approaches is challenging, but options such as combining NEO-MRCI with vibronic coupling theory^78^ or instanton theory^79,80^ are currently being explored.

**Figure 3:**
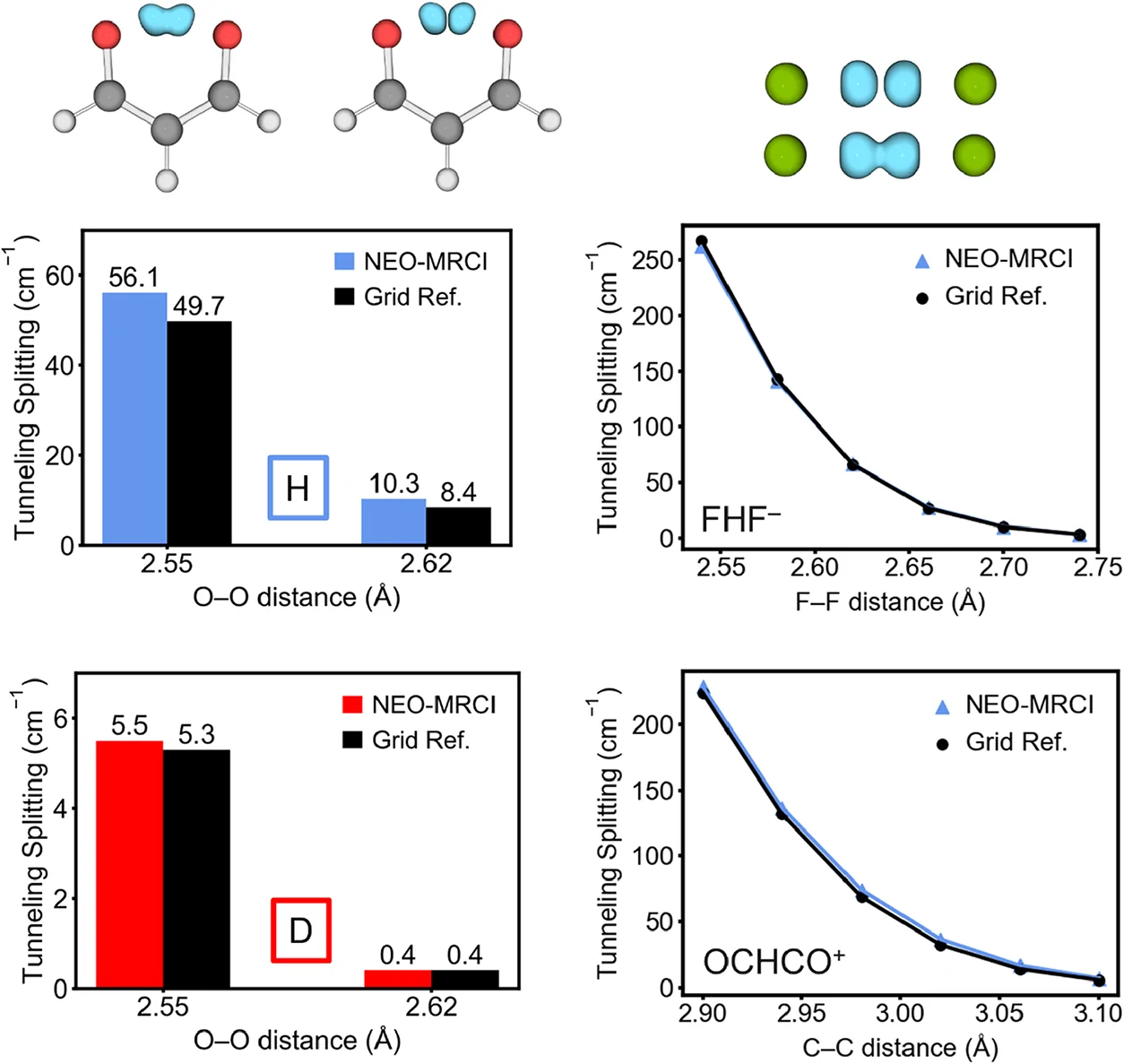
Hydrogen tunneling splittings for fixed geometries computed with NEO-MRCI compared to a numerically exact grid-based reference. The protonic densities for the lowest two vibronic states are shown in cyan for malonaldehyde (left) and FHF^–^ (right). The bar graphs for malonaldehyde show the results for the H and D tunneling splittings for two symmetric geometries associated with different O–O distances. The plots for the linear molecules on the right show the H tunneling splittings as functions of the indicated heavy-atom distances, where the C–O distance is kept fixed for OCHCO^+^. The NEO-MRCI calculations included all single excitations (i.e., single electron and single proton) and double electron–proton excitations. Adapted with permission from ref 77. Copyright 2025 American Chemical Society.

## NEO Density Functional Theory

### Multicomponent DFT

Although wave function methods such as NEO-CCSD­(T) and NEO-MRCI represent gold standards, the current workhorse for large chemical, biological, and materials systems is NEO-DFT. Multicomponent DFT refers to DFT with more than one type of particle, in this case electrons and protons.^38,65–69^ The multicomponent extensions of conventional electronic DFT were developed decades ago. The total energy is a functional of both the electronic and protonic densities, ρ^e^ and ρ^p^

4 $$E\left[\rho^{e},\rho^{p}\right]=E_{\mathrm{ext}}\left[\rho^{e},\rho^{p}\right]+E_{\mathrm{ref}}\left[\rho^{e},\rho^{p}\right]+E_{\mathrm{exc}}\left[\rho^{e}\right]+E_{\mathrm{pxc}}\left[\rho^{p}\right]+E_{\mathrm{epc}}\left[\rho^{e},\rho^{p}\right]$$

The first term represents the interaction of both densities with the external potential (i.e., the classical nuclei), and the second term represents the energy of the noninteracting reference system, including the kinetic energies of the electrons and protons and the classical Coulomb interactions among them. The third term is the electron–electron exchange–correlation functional, which is defined identically to the definition in conventional electronic DFT,^69^ allowing the use of existing electronic exchange–correlation functionals. The fourth term is the proton–proton exchange–correlation functional, which becomes trivial because exchange and correlation interactions between protons are negligible for molecular systems due to the localization of the protonic orbitals. As mentioned above, the proton–proton exchange terms are typically 8–10 orders of magnitude smaller than their electronic counterparts,^44^ and proton–proton correlation is also negligible.^44,58^ Thus, only the exact proton–proton self-exchange terms need to be included to avoid self-interaction error. The last term is the electron–proton correlation functional, which is extremely important due to the attractive interaction between an electron and a proton but has served as a great challenge in the field of multicomponent DFT.

To address this challenge, our group has expended significant effort to develop practical yet sufficiently accurate electron–proton correlation functionals.^81–84^ The most widely used electron–proton correlation functional in current applications within the NEO framework is the epc17-2 functional.^81,82^ This functional was inspired by the Colle–Salvetti formalism^85^ that produced the LYP electron–electron correlation functional.^86^ Starting with a multicomponent explicitly correlated electronic–nuclear wave function, we made a series of approximations, such as relating the inverse correlation length to the geometric mean of the inverse Wigner–Seitz radii for an electron and a proton,^81^ rather than simply the inverse Wigner–Seitz radius for an electron, which underlies the LYP functional. Our formalism also accounted for the opposite charges of the electron and proton. The final form depends on three parameters that were determined by fitting to proton densities and energies. The robustness of this strategy was validated by the development of a closely related electron–proton functional, epc18,^83^ which relates the inverse correlation length to the *arithmetic* mean of the inverse Wigner–Seitz radii for an electron and a proton and produces nearly identical results as those produced by epc17-2. In addition to these local density approximation functionals, we also developed a generalized gradient approximation electron–proton correlation functional, epc19,^84^ but this functional does not provide a significant improvement.

Note that previous functionals based on the Colle–Salvetti formalism assumed that the inverse correlation length depends on only the Wigner–Seitz radius for an electron (i.e., only on the electronic density).^87–90^ This assumption is not physically reasonable because the correlation length between two different types of particles should depend on both densities. Additionally, typically these previous functionals were added as corrections to the energies rather than being included during the self-consistent field procedure, leading to unphysically overlocalized proton densities and therefore unreliable properties such as optimized geometries, vibrational frequencies, and vibronic couplings. The epc17, epc18, and epc19 functionals utilize a correlation length dependent on both the electronic and protonic densities and are included during the self-consistent field procedure, thereby avoiding these shortcomings.^81–84^ More recently, other promising strategies for designing local hybrid electron–proton correlation functionals^91^ and multicomponent double-hybrid functionals^92^ have been explored.

Based on the energy functional given in eq ([Disp-formula eq4]) and a reference wave function of the form defined in eq ([Disp-formula eq1]), the variational method can be used to minimize the energy with respect to the electronic and nuclear orbitals. This procedure leads to electronic and nuclear Kohn–Sham equations of the form given in eq ([Disp-formula eq2]), except the Fock matrices are replaced by the Kohn–Sham matrices, which include electronic exchange–correlation and electron–proton correlation. As for NEO-HF calculations, the electronic and nuclear equations are solved self-consistently in NEO-DFT calculations, and the protonic basis function centers can be optimized variationally in conjunction with this self-consistent field procedure.

To illustrate the importance of electron–proton correlation, we have compared the proton densities with and without this correlation using both NEO-CCSD and NEO-DFT/epc17-2. Our calculations on molecules such as HCN and FHF^–^ show that the neglect of electron–proton correlation leads to proton densities that are much too localized, with the maximum magnitude for the normalized proton density around three times greater than that for a numerically exact grid-based reference. Inclusion of correlation via NEO-CCSD or NEO-DFT/epc17-2 for these molecules significantly delocalizes the proton density,^44,81,93^ leading to much better agreement with the numerically exact reference. Similarly, the ZPE associated with the quantum proton is typically around three times too high when electron–proton correlation is neglected^94–97^ and becomes much more accurate when electron–proton correlation is included via either NEO-CCSD or NEO-DFT/epc17-2. For example, the ZPE associated with the hydrogen-bonding proton for a phenoxyl-phenol complex is 25.40 kcal/mol without epc17-2 and 7.87 kcal/mol with epc17-2, compared to the reference value of 7.52 kcal/mol obtained from a numerically exact grid-based method.^95^ Similar trends in the ZPE with and without epc17-2 were found for [2,2′-bipyridyl]-3-ol,^94^ a tyrosine-glutamate model complex,^95^ and a series of more than 40 acids, including protonated nitrogen bases, nitrophenols, anilines, and diamines.^97^ As mentioned above, the most accurate anharmonic ZPE within the NEO framework is provided by NEO-CCSD­(T).^58^ These types of 200% errors in proton densities and energies when electron–proton correlation is neglected provide a clear indication that electron–proton correlation should be included in NEO calculations.

To make NEO-DFT practical for large chemical, biological, and materials systems, several algorithmic advances have been required. For example, the simultaneous optimization of both electronic and nuclear orbitals speeds up the self-consistent field procedure.^98^ In addition, utilizing the nuclear Hartree product representation^70^ for the quantum protons also significantly decreases the number of iterations required for convergence without adversely affecting accuracy.^71^ Other methods such as density fitting^55,60,99–101^ and Cholesky decomposition^102,103^ have substantially sped up the integral calculations, analogous to their roles in conventional electronic structure calculations. These methods have enabled NEO-DFT calculations on systems with hundreds of quantum protons. Additionally, the implementation of analytical gradients and analytical Hessians^104^ for NEO-DFT allows the calculation of equilibrium geometries, transition states, minimum-energy paths, and dynamics on the ground vibronic state. The remainder of this section presents developments that further extend the applicability of NEO-DFT, along with applications to interesting chemical systems.

### NEO-DFT Dynamics on the Ground Vibronic State

To simulate nuclear dynamics on the ground vibronic state, the classical nuclei can be propagated according to Newton’s equations of motion. In such molecular dynamics (MD) simulations, the forces are obtained from the gradient of the NEO ground vibronic-state energy. A challenge that arises in such simulations is that the protonic basis function centers must be able to move to account for the use of a finite protonic basis set. One strategy is to variationally optimize the protonic basis function center position at each MD time step. However, this strategy is computationally expensive, especially given that the protonic basis function center optimization must be converged tightly to enable acceptable energy conservation. As an alternative, more computationally efficient strategy, we developed the extended Lagrangian molecular dynamics (NEO-ELMD) method.^105^ In this strategy, the protonic basis function centers are treated as auxiliary degrees of freedom and are propagated via classical MD along with the classical nuclei. The mathematically conserved quantity in this case is the extended energy, which includes the kinetic energy of the protonic basis function centers as well as the system energy. We have shown that the NEO-ELMD approach agrees well with the more expensive variational optimization approach and conserves the extended energy to a high degree.^105^ Application of Lagrangian constraints equating each protonic basis function center with its corresponding proton position expectation value during the MD is mathematically equivalent to constrained NEO (CNEO) MD, which has been shown to be effective.^106,107^ These constraints have minimal impact on the dynamics for the systems studied to date but may be useful to ensure the stability of longer trajectories.

The NEO-ELMD method has been applied to several proton transfer reactions, showing that the quantization of transferring protons can alter both the rate and mechanism of such reactions.^105^ A double proton transfer reaction induced by either electrochemical or photochemical oxidation^108,109^ is shown in Figure 4A. Starting at the equilibrium geometry of the neutral molecule, electrochemical or photochemical oxidation is modeled by instantaneously removing an electron, resulting in a cationic species. The NEO-ELMD dynamics following this oxidation exhibit asynchronous double proton transfer: first, a proton transfers from the oxygen on phenol to the nitrogen on imidazole, and then a proton transfers from the other nitrogen on imidazole to the nitrogen on pyridine. Starting with the same initial conditions, the second imidazolic proton transfer does not occur with a conventional classical treatment of the transferring protons, highlighting the importance of the nuclear quantum effects captured by the NEO method. The asynchronicity of the double proton transfer observed in the NEO-ELMD dynamics is not observable on the time scale of the electrochemical experiments performed on these types of molecules^108^ but rather would require more challenging ultrafast spectroscopy experiments.^109^ Thus, these types of nonequilibrium MD simulations, which include the nuclear quantum effects associated with the transferring protons, provide mechanistic insights that are not easily accessible experimentally.

**Figure 4:**
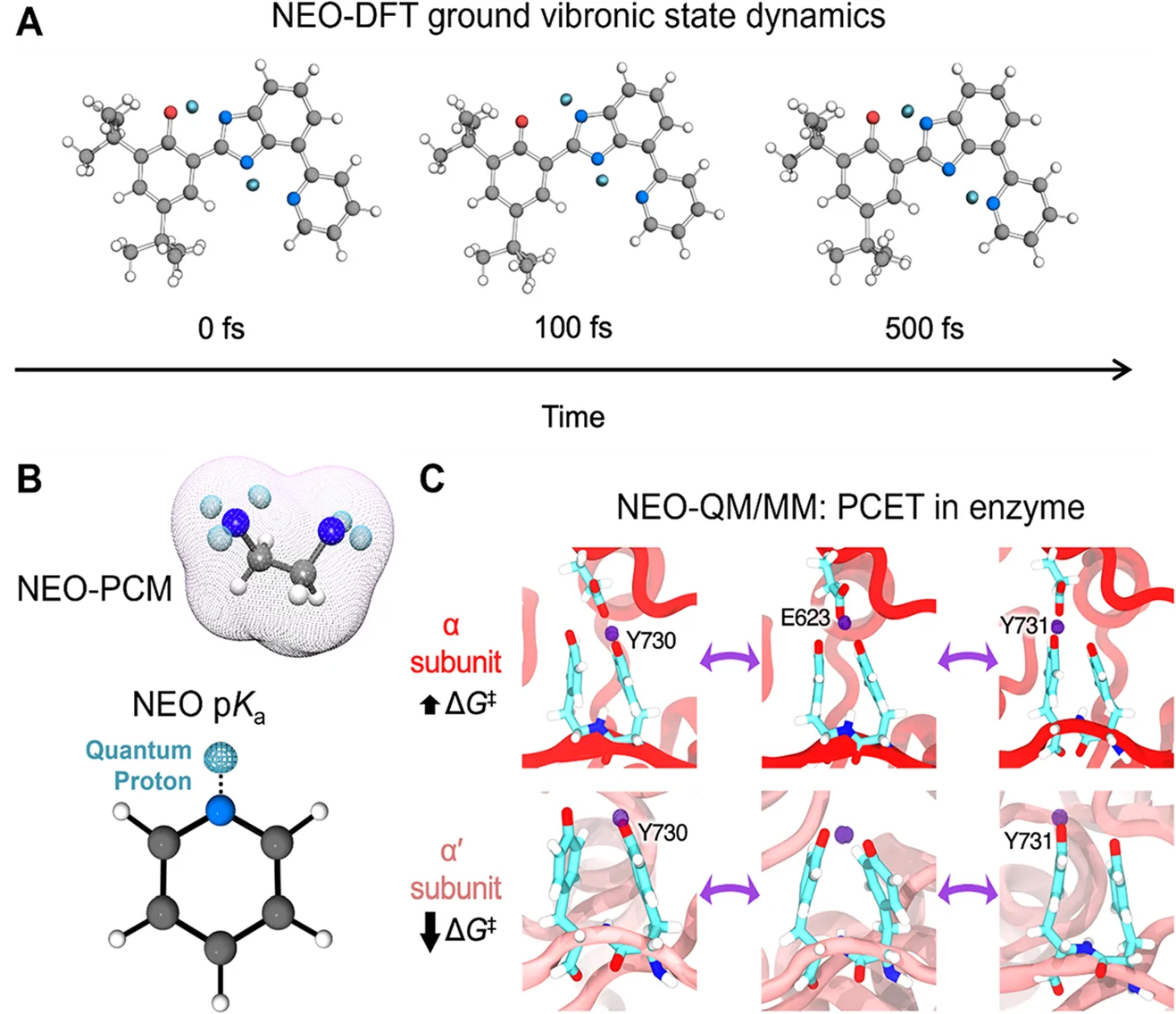
Applications of NEO-DFT to chemical and biological systems. (A) Dynamics of electrochemically or photochemically induced PCET on the NEO-DFT ground vibronic state. The two transferring protons are treated quantum mechanically, with their proton densities shown in cyan. The double proton transfer is asynchronous: the first proton transfers from the phenol oxygen to the imidazole nitrogen, and then the second proton transfers from the other imidazole nitrogen to the pyridine nitrogen. (B) Depiction of a molecular-shaped cavity used in the NEO-PCM method for continuum solvation, which is used in a protocol for calculating p*K*_a_ values that include the anharmonic effects of the quantized protons shown in cyan. (C) NEO-QM/MM free energy simulations of PCET in the enzyme ribonucleotide reductase, where the explicitly shown atoms are treated quantum-mechanically with NEO-DFT. The transferring proton is quantized with its proton density shown in purple. Quantization of the proton slightly increases the free energy barrier in the α subunit due to ZPE effects and significantly decreases the free energy barrier in the α′ subunit due to a combination of ZPE and shallow hydrogen tunneling effects, as indicated by the bilobal proton density in the lower middle frame. Panel (A) reproduced from ref 105; licensed under a Creative Commons Attribution (CC BY) license. Panel (C) reproduced with permission from ref 95. Copyright 2024 American Chemical Society.

### NEO-DFT in a Solution or Enzyme Environment

To move beyond the gas phase, we have extended NEO methods to include a complex environment such as a solvent or protein. A variety of continuum solvation formalisms, including the polarizable continuum model (PCM),^110^ have been implemented in conjunction with NEO-DFT. In continuum solvation models, the solute molecule is embedded in a cavity, and the solvent is described as a uniform dielectric continuum. The NEO-PCM method,^111,112^ as well as other NEO continuum solvation formalisms,^113^ includes mutual polarization between the solvent and both the electronic and protonic densities. To incorporate a heterogeneous environment, we developed NEO quantum mechanical/molecular mechanical (QM/MM) approaches.^114^ In these approaches, the QM region is treated quantum mechanically with a method such as NEO-DFT, and the remainder of the system is treated with a molecular mechanical force field. Within the QM region, either all protons or only certain specified protons may be treated quantum mechanically. Using electrostatic embedding, the partial charges associated with the molecular mechanical environment are included as an external potential in the solution of the NEO-DFT equations, allowing the electronic and protonic densities to respond to the protein environment. The NEO-PCM and NEO-QM/MM methods can be used to calculate single-point energies or to optimize geometries. We have also combined these methods with MD approaches, where the classical nuclei can move in either a continuum solvent environment or a heterogeneous molecular mechanical environment.

An important application of NEO-PCM is the calculation of solution-phase p*K*_a_ values.^97^ Continuum solvation models have been used extensively to compute p*K*_a_ values with conventional electronic structure methods.^115–117^ Typically, these approaches use a normal mode analysis based on the harmonic approximation of vibrational modes to compute the ZPE. We have devised a general protocol for computing relative p*K*_a_ values using the NEO-DFT-PCM method to treat the key proton(s) quantum mechanically (cyan protons in Figure 4B).^97^ The anharmonic ZPE associated with these quantum protons is inherently included in the NEO-DFT energy. The ZPE associated with the other nuclei and the vibrational thermal and entropic contributions of all nuclei were treated within the harmonic approximation. Although only a slight improvement over the fully harmonic vibrational treatment was observed for simple acids, the NEO protocol offers a notable improvement for hydrogen-bonded acids, where anharmonic effects are more pronounced.^97^ The NEO framework provides a practical strategy for incorporating anharmonic effects into p*K*_a_ predictions in a computationally efficient and straightforward manner.

We used the NEO-QM/MM method to simulate proton-coupled electron transfer (PCET) in the enzyme ribonucleotide reductase.^95^ Our work has shown that PCET between two specific tyrosine residues, Y731 and Y730, proceeds on the vibronic ground state. We simulated this reaction in two different protein subunits, α and α′. In the α subunit, a glutamate residue, E623, has been shown to be able to mediate this PCET reaction by hydrogen bonding to the transferring proton as it moves between the two tyrosine oxygen atoms (Figure 4C). Initially, we simulated this reaction using conventional QM/MM methods,^118^ where the transferring hydrogen nucleus was treated classically, in conjunction with the finite temperature string method with umbrella sampling.^119^ This approach includes conformational sampling of the solvated enzyme and thereby produces multidimensional free energy surfaces. Subsequently, we performed these calculations using NEO-DFT instead of DFT to enable quantization of the transferring proton in the QM region, which was composed of Y730 and Y731, as well as E623 for the α subunit.^95^ We devised a simple reweighting procedure to obtain the NEO-DFT free energy surfaces from the conventional DFT sampling, although direct NEO-DFT QM/MM free energy simulations should give the same results with adequate sampling.

The resulting NEO-DFT free energy surfaces inherently include the ZPE and shallow tunneling effects of the transferring proton.^95^ In the α subunit, quantizing the proton increases the free energy barrier by around 0.8 kcal/mol. This finding is surprising because nuclear quantum effects are usually expected to decrease the free energy barrier. This unusual finding arises from the mediation of proton transfer by E623 because the ZPE for the proton near glutamate is slightly higher than the ZPE for the proton near tyrosine. In the α′ subunit, quantizing the proton decreases the free energy barrier by 4–5 kcal/mol due to a combination of ZPE and shallow tunneling effects (see bilobal purple proton density in Figure 4C). Such a decrease in the free energy barrier has a significant impact on the rate constant for this PCET reaction. This example illustrates that nuclear quantum effects can either raise or lower the free energy barrier, thereby either decreasing or increasing the rate constant and leading to a range of possible kinetic isotope effects. The NEO-DFT QM/MM method provides an efficient route for incorporating nuclear quantum effects into simulations of large-scale biological systems.

### Periodic NEO-DFT

Periodic NEO-DFT enables the investigation of extended systems such as solids and surfaces.^120^ Our implementation in the FHI-aims software package^49^ utilizes numerical atom-centered orbitals for the electronic basis set and Gaussian protonic basis sets.^50^ A variety of electronic exchange–correlation functionals, as well as the epc17-2 electron–proton correlation functional,^81,82^ are available. Our initial applications included a hydrogen boride sheet and a titanium oxide–water interface.^120^ More recently, we used periodic NEO-DFT to investigate isotope effects for H/D adsorption, OH^–^/OD^–^ adsorption, and H_2_O/D_2_O monolayers on Pt(111) surfaces.^96^ The CNEO-DFT method^121^ and a multicomponent cluster-based correction to periodic DFT^122^ have also been used to study nuclear quantum effects at Pt(111) surfaces but in the absence of electron–proton correlation. Another recent application of periodic NEO-DFT is our investigation of nuclear quantum effects in high-pressure superconducting hydrides and ice.^123^ The remainder of this subsection summarizes the results for these applications.

In our study of H/D adsorption on Pt(111), we performed geometry optimizations to determine the lowest-energy adsorption site.^96^ We considered all three possible adsorption sites: the top site and the fcc and hcp hollow sites (Figure 5A). Using conventional DFT without ZPE, the top site is lowest in energy. Using NEO-DFT, which inherently includes the ZPE of the adsorbing H or D, the fcc hollow site is lowest in energy, which is consistent with experimental measurements.^124,125^ If the ZPE is added to the conventional DFT electronic energy using a normal mode analysis based on a phonon calculation, the fcc hollow site is also lower in energy. However, phonon calculations are typically more expensive than periodic NEO-DFT energy calculations and rely on the harmonic approximation, whereas NEO-DFT includes anharmonic effects. Moreover, beyond identifying the energetically favored adsorption site, NEO-DFT provides additional information about geometric isotope effects. Using conventional DFT, the same minimum-energy geometry is obtained with H or D, but using NEO-DFT, different geometries are obtained because the hydrogen density is more delocalized than the deuterium density. In particular, we found that the Pt–H distance is ∼0.004 Å greater than the Pt–D distance. We were also able to apply an external electric field and an accompanying dipole correction to mimic a potential bias at the Pt(111) surface. We found that the energetically favored adsorption site changes from the fcc hollow site to the top site for both H and D upon application of a sufficiently large field. This finding is consistent with experiments and calculations indicating that the fcc hollow site is favored for underpotential deposition, whereas the top site is favored for overpotential deposition.^125,126^

**Figure 5:**
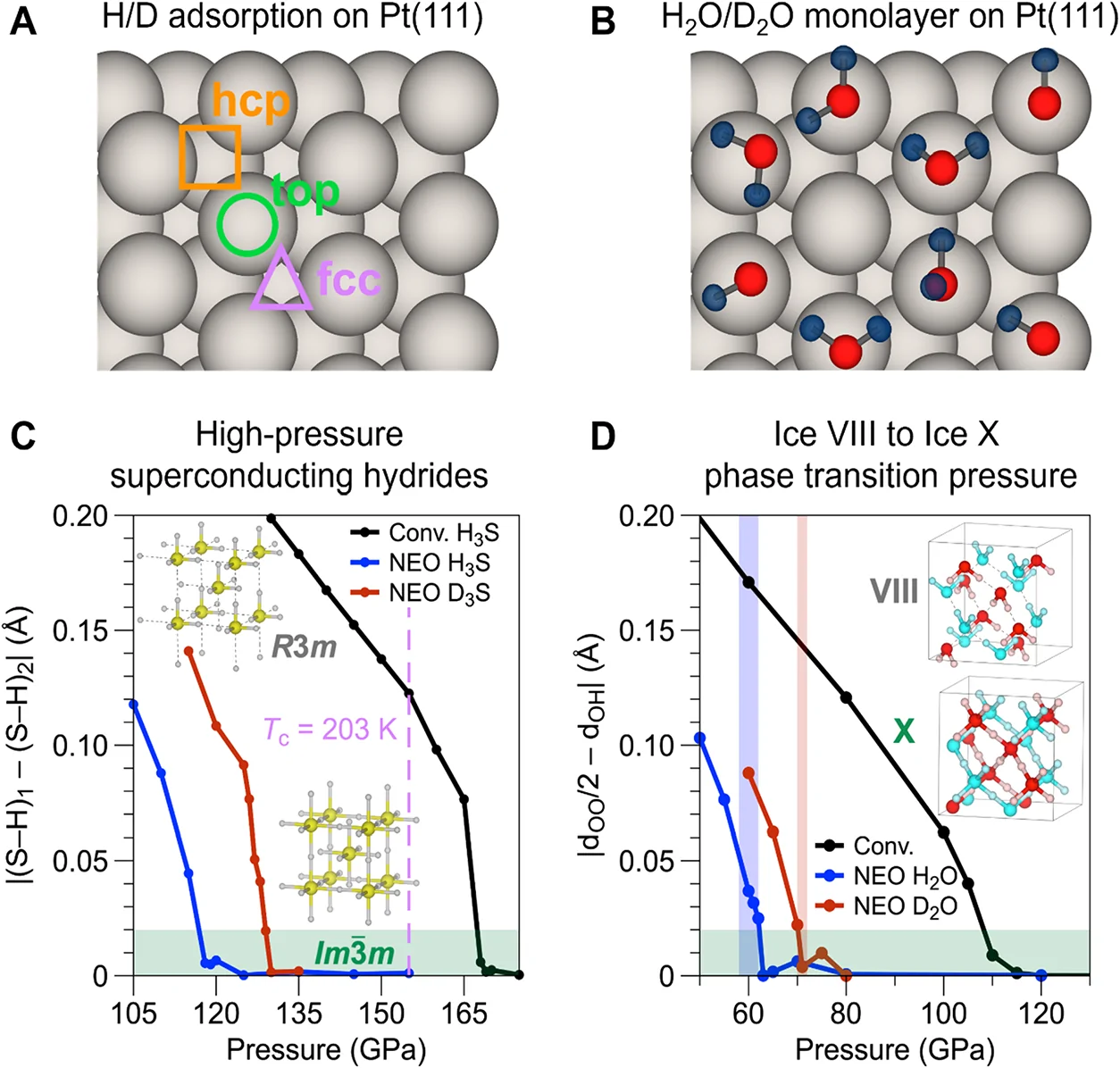
Applications of periodic NEO-DFT to surfaces and solids. (A) Periodic NEO-DFT geometry optimizations of H and D adsorption on Pt(111) correctly identify the fcc hollow site as the energetically favored site and elucidate geometric isotope effects. (B) Periodic NEO-DFT geometry optimizations of H_2_O and D_2_O monolayers on Pt(111) provide structural information about interfacial water as well as geometric isotope effects. (C) Periodic NEO-DFT geometry optimizations of H_3_S and D_3_S under high pressure predict symmetrization pressures consistent with experiment (purple dashed line indicates pressure corresponding to highest experimental *T*_c_) and with calculations using the more expensive SSCHA method. (D) Periodic NEO-DFT geometry optimizations of H_2_O and D_2_O under high pressure predict phase-transition pressures in agreement with experimental data (shaded blue and red regions) and more expensive computational methods. For both the superconducting hydrides and the ice phase transition, conventional periodic DFT calculations predict transition pressures that are much too high (black curves, compared to blue and red curves computed with NEO-DFT), showing that nuclear quantum effects are essential for these types of systems. Panels (A) and (B) adapted with permission from ref 96. Copyright 2025 American Chemical Society. Panel (C) adapted with permission from ref 123. Copyright 2026 National Academy of Sciences.

We also used periodic NEO-DFT to optimize H_2_O and D_2_O hexagonal monolayer structures on the Pt(111) surface (Figure 5B).^96^ We found that the average distance between the oxygen atoms and the Pt(111) surface is smaller for H_2_O than for D_2_O, indicating a stronger interaction of H_2_O with the surface. Furthermore, the average O–D covalent bond is ∼0.005 Å shorter than the average covalent O–H bond. This distance change is small but is validated by the experimentally measured decrease of the O–H bond of gas phase water by 0.0038 Å upon deuteration.^127^ Such a geometric isotope effect is not accessible via conventional DFT geometry optimizations. We also observed weaker hydrogen-bonding interactions within the monolayer for D_2_O than for H_2_O. The interaction of interfacial water with electrode surfaces is known to play a critical role in electrochemistry. Understanding the interactions of H and H_2_O with a metal electrode surface both with and without an applied potential is important for designing and controlling electrocatalytic processes.

Another exciting application of periodic NEO-DFT focuses on hydrogen-rich materials.^123^ In our investigation of high-pressure superconducting hydrides, we performed periodic NEO-DFT geometry optimizations in the presence of applied pressure. The H_3_S and D_3_S systems are thought to be high-temperature (high-*T*_c_) superconductors in the hydrogen-bond symmetrized phase, i.e., when the S–H distances for shared hydrogens become nearly equivalent (Figure 5C, green shaded region).^13^ High-*T*_c_ superconductivity has been observed experimentally at a range of pressures, including 155 GPa (Figure 5C, purple dashed line) as well as lower pressures.^128^ Conventional periodic DFT calculations predict a hydrogen-bond symmetrization pressure that is much too high (Figure 5C, black curve), predicting the incorrect phase at pressures that have been shown experimentally to exhibit superconductivity. In contrast, periodic NEO-DFT calculations predict a much lower hydrogen-bond symmetrization pressure (Figure 5C, blue and red curves), predicting the correct symmetric phase at 155 GPa. Thus, the periodic NEO-DFT symmetrization pressure is consistent with experimental measurements of high-*T*_c_ superconductivity. Moreover, the symmetrization pressure and H/D isotopic shift predicted by periodic NEO-DFT are consistent with those predicted by the more expensive stochastic self-consistent harmonic approximation (SSCHA) method for incorporating nuclear quantum effects.^13^ The periodic NEO-DFT method can also be used to calculate phonon spectra,^129^ opening up many exciting directions in the field of superconductivity.

We have found similar behavior for calculations of the ice VIII to ice X phase-transition pressure. In this case, the ice X phase corresponds to a symmetric hydrogen-bonding structure (Figure 5D, green shaded region).^130^ Again, conventional periodic DFT predicts a phase-transition pressure that is much too high, around 110 GPa (Figure 5D, black curve). Periodic NEO-DFT predicts phase-transition pressures for H_2_O and D_2_O in agreement with experimental measurements (Figure 5D, compare blue and red curves to shaded blue and red regions). For H_2_O, the NEO-DFT transition pressure is 62 GPa, compared to the experimental range of 58–62 GPa,^131–134^ and for D_2_O, the NEO-DFT transition pressure is 71 GPa, compared to the experimental range of 70–72 GPa.^132,135^ This high level of quantitative agreement with experiment may be due to a balance among the various approximations. The phase-transition pressures predicted by periodic NEO-DFT are also consistent with prior results^12^ obtained using the much more expensive SSCHA method and a path integral molecular dynamics method for incorporating nuclear quantum effects using the same PBE electronic functional.^136^ Periodic NEO-DFT is clearly capturing the nuclear quantum effects in high-pressure materials in a physically meaningful manner at a relatively low computational cost.

### NEO Multistate DFT for Describing Hydrogen Tunneling and Kinetic Isotope Effects

A limitation of NEO-DFT with currently available electron–proton correlation functionals is that it is unable to describe deep tunneling, although it can describe some shallow tunneling effects (Figure 4C, bilobal purple proton density). As discussed above, hydrogen tunneling is often treated as a multireference problem within the NEO framework and can be described effectively using the NEO-MRCI method,^64^ which provides quantitatively accurate hydrogen tunneling splittings for fixed geometries (Figure 3).^77^ To enable the description of hydrogen tunneling within the NEO-DFT framework, we developed the NEO multistate DFT (NEO-MSDFT) method,^137,138^ inspired by conventional electronic MSDFT.^139,140^ As for the NEO-MRCI implementation for hydrogen tunneling, the transferring proton is represented by two protonic basis function centers. Even for a symmetric system, the NEO-DFT solution is localized near either the donor or the acceptor atom using available electron–proton correlation functionals. NEO-MSDFT combines these two localized NEO-DFT states with a nonorthogonal configuration interaction scheme, leading to delocalized, bilobal proton densities for symmetric or nearly symmetric systems (see proton densities for malonaldehyde in Figure 3). Two parameters are introduced to account for limitations of the electron–proton correlation functional and approximations underlying the NEO-MSDFT approach. These parameters are obtained by an analysis of small model systems^137^ and then are fixed for subsequent calculations, similar to the parameters within density functionals. Although it is not parameter-free, NEO-MSDFT is much more computationally efficient and straightforward to use compared to NEO-MRCI.

The NEO-MSDFT method is particularly useful for describing hydrogen transfer reactions, including proton, hydride, and hydrogen atom transfer. NEO-MSDFT has also been generalized to an arbitrary number of quantum protons to allow for the description of systems involving the transfer and tunneling of multiple hydrogen nuclei.^138^ We have shown that NEO-MSDFT predicts hydrogen tunneling splittings for single and double proton transfer at fixed geometries in quantitative agreement with numerically exact grid-based calculations.^137,138^ Moreover, the implementation of analytical gradients for NEO-MSDFT enables geometry optimizations and molecular dynamics simulations of both single and multiple proton transfer reactions.^141–143^ For the direct dynamics of hydrogen tunneling in malonaldehyde, where the classical nuclei are propagated on the ground vibronic state, the appearance of a bilobal proton density is indicative of hydrogen tunneling (Figure 6A).^142^ Nonadiabatic dynamics methods,^144,145^ such as Ehrenfest^146^ and surface hopping^147^ dynamics, can be used to propagate the classical nuclei over multiple NEO-MSDFT vibronic surfaces, thereby providing more accurate descriptions of hydrogen tunneling.^142^ In addition to the prototypical hydrogen tunneling reaction in malonaldehyde, such methods have been used to simulate double proton transfer in the formic acid dimer and the heterodimer of formamidine and formic acid.^143^

**Figure 6:**
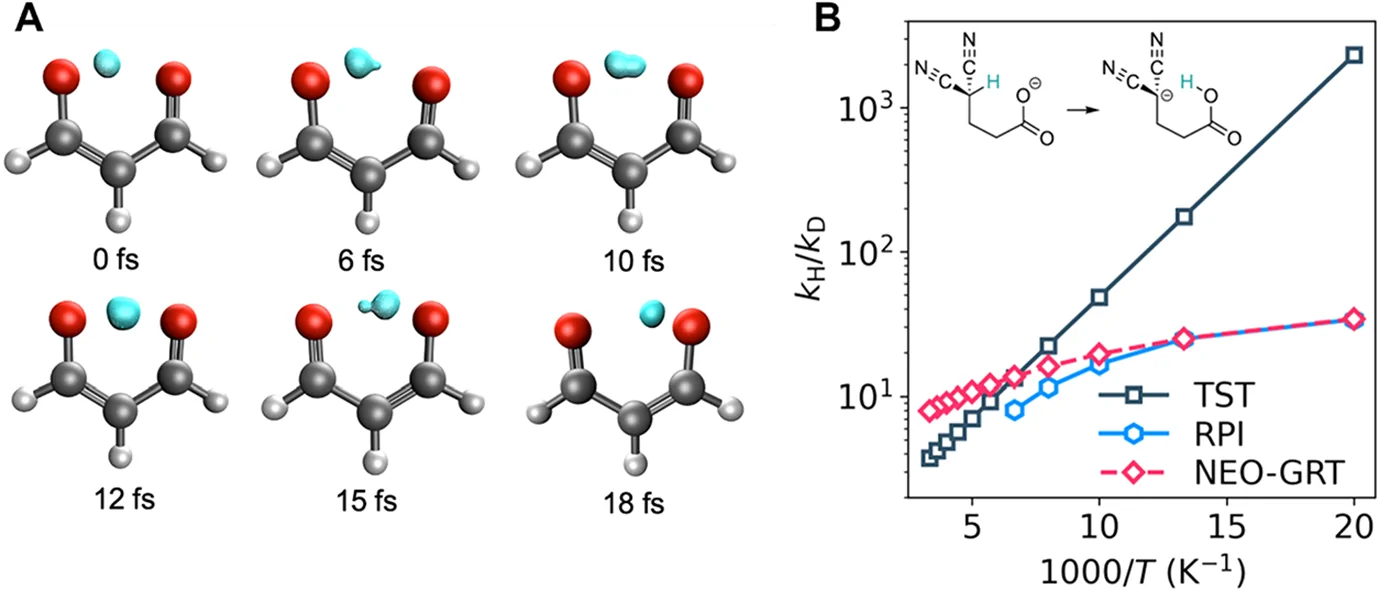
Applications of NEO-MSDFT to hydrogen tunneling systems. (A) Hydrogen tunneling dynamics on the NEO-MSDFT ground vibronic surface for malonaldehyde, where the bilobal character of the proton density indicates tunneling. (B) Kinetic isotope effects versus inverse temperature for a gas-phase molecular system in the deep tunneling regime. NEO general rate theory (GRT) results are in excellent agreement with ring-polymer instanton (RPI) theory results at low temperatures, whereas transition state theory (TST) is qualitatively incorrect due to the neglect of tunneling. Figures adapted with permission from refs 142 and 148. Copyright 2022 and 2026 American Chemical Society.

Recently, we developed a NEO general rate theory (NEO-GRT) for hydrogen transfer and showed that it can predict hydrogen kinetic isotope effects in the deep tunneling regime.^148^ This theory is based on a hydrogen transfer rate constant expression that spans the vibrationally adiabatic and nonadiabatic tunneling regimes.^149–151^ Our initial implementation of this general rate theory uses NEO-MSDFT to compute the vibronic coupling, which strongly influences the kinetic isotope effect. We found that the kinetic isotope effects computed with NEO-GRT agree with those calculated using ring-polymer instanton theory^79,80^ for full-dimensional molecular systems studied at the same level of electronic structure theory (Figure 6B). Further developments are underway to enable kinetic isotope effect calculations in solution at higher temperatures. Note that a recently developed constrained NEO-DFT-based transition state theory^152^ provides rate constants for reactions with minimal tunneling at room temperature but cannot capture significant tunneling effects. Because NEO-GRT captures deep hydrogen tunneling effects at a relatively low computational cost, NEO-GRT is a promising approach for predicting kinetic isotope effects in a wide range of organic, inorganic, and biological systems.

### Linear-Response TDDFT

We developed linear-response NEO-TDDFT to describe excited states within the multicomponent DFT framework.^153^ Analogous to conventional electronic linear-response TDDFT,^154^ this method is based on the linear response of the system to perturbative electric fields. The form of the matrix equation to be solved is the same as that for conventional electronic TDDFT, but the matrices are expanded to include the nuclear contributions and couplings between the electronic and nuclear contributions. The solution of this equation provides electronic, vibrational, and vibronic excitation energies in a single calculation. As for conventional electronic TDDFT, only single excitations can be described within this implementation. We have shown that the NEO-TDDFT proton vibrational excitation energies are accurate when compared to other types of anharmonic vibrational frequency calculations as long as sufficiently large electronic basis sets are used.^155^ We have also implemented analytical gradients, allowing geometry optimizations and excited-state dynamics using NEO-TDDFT.^94^ We used these methods to compute 0–0 adiabatic excitation energies, where the anharmonic ZPE associated with the quantum protons is included inherently in the NEO-TDDFT excitation energy, and the ZPE associated with the classical nuclei is computed using seminumerical NEO Hessians within the harmonic approximation.^94^ In principle, nonadiabatic dynamics simulations using Ehrenfest or surface hopping methods could be performed for the classical nuclei moving on the NEO-TDDFT vibronic surfaces, although nonadiabatic coupling vectors would need to be implemented. Linear-response NEO-TDDFT enables a wide range of applications to excited-state processes with quantized protons.

## Real-Time NEO Methods

A primary objective of the NEO approach is to simulate the real-time nuclear–electronic dynamics of chemical and photochemical systems. One strategy is to propagate the classical nuclear dynamics on the vibronic surfaces either adiabatically, on a single vibronic surface, or nonadiabatically, over multiple vibronic surfaces. In the previous sections, we have discussed NEO-DFT dynamics on the ground vibronic state for a double proton transfer reaction (Figure 4A) and NEO-MSDFT dynamics on the ground vibronic state for a hydrogen tunneling process (Figure 6A). We have also discussed the option to perform nonadiabatic dynamics over multiple NEO-MSDFT or NEO-TDDFT vibronic surfaces using Ehrenfest or surface hopping methods. These methods are based on the premise that the classical nuclei move on adiabatic vibronic energy surfaces generated by using NEO-DFT, NEO-MSDFT, or NEO-TDDFT to solve the nuclear–electronic *time-independent* Schrödinger equation at each classical nuclear configuration.

An alternative strategy is to solve the nuclear–electronic *time-dependent* Schrödinger equation to directly propagate the electronic and nuclear densities in time. To distinguish such methods from those based on adiabatic vibronic surfaces, they are denoted “real-time” NEO methods, analogous to real-time time-dependent electronic structure methods.^156^ Using this strategy, we have developed real-time NEO-TDDFT (RT-NEO-TDDFT)^157^ and NEO time-dependent configuration interaction (NEO-TDCI).^158^ These methods have opened up a wide range of applications, including photoinduced proton transfer, vibrational spectroscopy, hydrogen tunneling dynamics, plasmon-induced chemistry, and polaritonic chemistry. This section will briefly describe the methods along with some of the most exciting applications.

### Real-Time NEO-TDDFT

To obtain the RT-NEO equations,^157^ we substitute the NEO-HF wave function given in eq ([Disp-formula eq1]) into the time-dependent Schrödinger equation, leading to the electronic and nuclear time-dependent Hartree–Fock equations

5 $$\begin{array}{l} i\hbar \frac{\partial }{\partial t}C^{e}\left(t\right)=F^{e}\left(t\right)C^{e}\left(t\right)\\ i\hbar \frac{\partial }{\partial t}C^{p}\left(t\right)=F^{p}\left(t\right)C^{p}\left(t\right) \end{array}$$

For RT-NEO-TDDFT, the Fock matrices are replaced with the Kohn–Sham matrices, which include electronic exchange–correlation and electron–proton correlation. Analogous to the time-independent equations given in eq ([Disp-formula eq2]), these time-dependent electronic and nuclear equations are strongly coupled to each other and must be solved in a self-consistent manner. In practice, these equations are transformed to von Neumann equations, which are propagated numerically in time. The classical nuclei can also be propagated numerically according to Newton’s equations of motion using forces obtained from the instantaneous NEO wave function.^159^ The nonadiabatic effects between the electrons and quantum protons are included within the RT-NEO-TDDFT equations, and the nonadiabatic effects between the classical and the quantum subsystems are included using Ehrenfest dynamics.^159,160^

A challenge that arises for RT-NEO-Ehrenfest dynamics is again related to the movement of the protonic basis function centers. Fixed protonic basis function centers with a finite basis set restrict the proton density to the spatial region spanned by the basis functions. Allowing the protonic basis function center to move enables the proton density to reach any region of space during dynamics. To address this challenge, several variants of a traveling proton basis (TPB) have been developed.^34,159–161^ Building upon the earlier variants, the most accurate and stable approach to date is the constrained traveling proton basis (c-TPB) approach.^34^ In this approach, the protons are treated as distinguishable using the nuclear Hartree product representation,^70^ which has been validated as discussed above.^71^ The classical nuclei and protonic basis function centers are propagated according to Newton’s equations of motion, assigning the proton mass for the protonic basis function centers. A Lagrangian constraint is applied to ensure that each protonic basis function center coincides with its corresponding proton position expectation value. Within this framework, we used an extended Lagrangian formulation to derive the RT-NEO-TDDFT c-TPB equations, which include an additional term in the von Neumann equations. The mathematically conserved quantity is the total energy of the system, including the kinetic energy associated with the protonic basis function centers. We have shown that this c-TPB method produces accurate nuclear–electronic quantum dynamics, conserves energy, and is computationally efficient.^34^ The powerful combination of RT-NEO-TDDFT with Ehrenfest dynamics and the c-TPB approach enables the simulation of spectra as well as nonequilibrium nuclear–electronic quantum dynamics. However, such Ehrenfest dynamics are unable to accurately describe the branching of disparate vibronic surfaces.

### RT-NEO-TDDFT for Spectroscopy

In real-time NEO-TDDFT for spectroscopy, typically a small perturbation is applied at the beginning of a RT-NEO-Ehrenfest dynamics simulation. The perturbation could be in the form of a small delta pulse applied to the system or a small distortion in the initial geometry. The spectra are calculated from the Fourier transform of the time-dependent dipole moment along the dynamical trajectory. The electronic spectrum can be obtained from the electronic dipole moment, the proton vibrational spectrum can be obtained from the protonic dipole moment, and the electronic and vibrational spectra can be computed from the total dipole moment. For simple molecules such as HCN, HNC, and FHF^–^, we have confirmed that the RT-NEO-TDDFT excitation energies are consistent with the linear-response NEO-TDDFT excitation energies.^159^

For more challenging vibrational spectra, we have employed a quasiclassical trajectory approach^162–164^ for generating initial conditions that include the ZPE of the classical nuclei.^165^ This approach enables sampling of the anharmonic regions of the vibronic surfaces and thus can capture the anharmonic heavy-atom nuclear motion, as well as the anharmonicity of the quantum protons that is inherently included in RT-NEO-TDDFT. Application of this approach to the formate-water complex (CHO_2_^–^·H_2_O) showcases its capability to capture distinct spectral features that are challenging for classical treatments.^165^ For example, the spectra obtained from RT-NEO-Ehrenfest dynamics captured the red-shifted and broadened OH stretch band due to strong anharmonicity arising from hydrogen bonding as well as coupling between motions of the hydrogen nuclei and the heavy nuclei. The combination of RT-NEO-TDDFT and Ehrenfest dynamics will assist in the interpretation of experimentally measured vibrational spectra and provide insights into the coupling between the vibrational modes.

### RT-NEO-TDDFT for Nonequilibrium Dynamics Induced by Photoexcitation

We have also applied RT-NEO-TDDFT to nonequilibrium excited-state intramolecular proton transfer in *o*-hydroxybenzaldehyde (*o*HBA).^160^ The π–π* excitation of this molecule was modeled by moving one electron from the highest occupied molecular orbital to the lowest unoccupied molecular orbital. This excitation induces proton transfer between the donor and acceptor oxygen atoms (Figure 7A). We treated the transferring proton quantum mechanically using the RT-NEO-TDDFT approach with either fixed classical nuclei or using Ehrenfest dynamics to propagate the classical nuclei. Our simulations of *o*HBA in the gas phase showed that quantizing the transferring proton increases the rate of proton transfer (Figure 7B),^34^ mainly due to delocalization of the proton, and slightly increases the kinetic isotope effect.^160^ We also performed these simulations in an implicit solvent using RT-NEO-PCM.^111^ In addition, we simulated this reaction in an explicit aqueous solvent using RT-NEO-Ehrenfest dynamics within a QM/MM framework (Figure 7D),^166^ where the *o*HBA was treated quantum mechanically and the explicit water molecules were treated with a molecular mechanical force field. All classical nuclei, including the solute and solvent, were propagated on the instantaneous vibronic surface. We found that the presence of explicit water can either accelerate or decelerate proton transfer, depending on the initial conditions.

**Figure 7:**
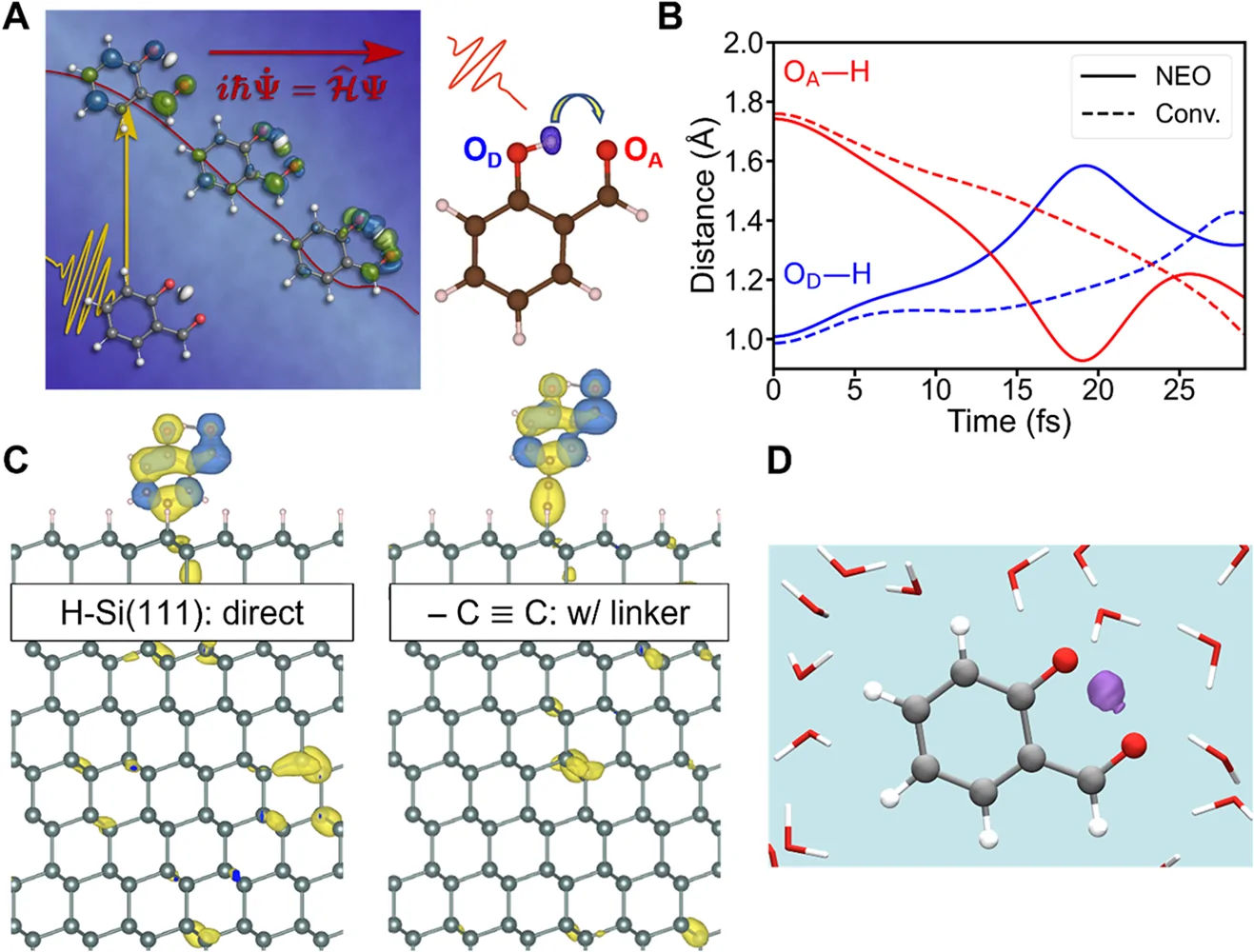
Applications of RT-NEO-TDDFT to excited-state intramolecular proton transfer in *o*HBA following π–π* excitation. (A) Snapshots along the RT-NEO-TDDFT trajectory in the gas phase with fixed classical nuclei, where the delocalized proton density is shown in gray. (B) Comparison of RT-NEO-TDDFT Ehrenfest dynamics with a quantized transferring proton and conventional RT-TDDFT Ehrenfest dynamics calculations. The distance between the transferring proton position expectation value and the donor or acceptor oxygen is plotted for the RT-NEO trajectory, and the same quantities with the classical proton position are plotted for the conventional trajectory. Quantization of the transferring proton increases the rate of proton transfer. (C) Periodic RT-NEO-TDDFT dynamics of *o*HBA attached to a Si(111) surface directly or with a linker showed that direct attachment fully suppresses proton transfer, and attachment via a linker slows down proton transfer. (D) RT-NEO-TDDFT Ehrenfest dynamics using a QM/MM approach with explicit water showed that the solvent can either accelerate or decelerate proton transfer, depending on initial conditions (proton density shown in purple). Panel (A) adapted with permission from ref 157. Copyright 2020 American Chemical Society. Panel (B) adapted with permission from ref 34; licensed under a Creative Commons Attribution (CC BY) license. Conventional data obtained with permission from ref 160. Copyright 2021 American Chemical Society. Panel (C) adapted with permission from ref 167. Copyright 2023 American Physical Society. Panel (D) adapted with permission from ref 166. Copyright 2023 American Chemical Society.

We also extended RT-NEO-TDDFT to periodic systems to enable the study of nuclear–electronic quantum dynamics in complex heterogeneous systems.^167^ To investigate the impact of a surface on photoinduced proton transfer dynamics, we attached *o*HBA to a Si(111) surface, either directly or through a linker (Figure 7C).^167^ In these calculations, all classical nuclei were fixed, although Ehrenfest dynamics is implemented for periodic systems. For the direct attachment, proton transfer is fully suppressed, mainly due to excited-state electron transfer from *o*HBA to the silicon surface. For the attachment via a linker, proton transfer still occurs but is slowed down relative to its rate for an isolated molecule in the gas phase, mainly due to a small percentage of excited-state electron transfer to the silicon surface. These simulations show that the environment, whether explicit solvent or a solid-state surface, can impact the dynamics of photoinduced proton transfer. Moreover, a quantum mechanical treatment of the transferring proton is essential for describing the effects of nuclear delocalization and, in some cases, shallow tunneling. More recently, we have used these RT-NEO methods to study single and double proton transfer in other molecules.^34^

Another exciting field that can be explored with RT-NEO methods is plasmonic chemistry, which utilizes collective electronic oscillations to drive reactions. Within this field, we have applied RT-NEO-TDDFT to plasmon-driven H_2_ photodissociation. Specifically, we studied H_2_ photodissociation near an Al_13_^–^ cluster (Figure 8).^168^ The process was initiated by applying a Gaussian pulse to excite the plasmonic mode of the Al_13_^–^ cluster, with an explicit treatment of all electrons of the system. The resulting plasmonic oscillations inject hot electrons into the antibonding orbital of H_2_, thereby inducing H_2_ dissociation. In the RT-NEO simulations, the hydrogen nuclei were treated quantum mechanically, and all aluminum nuclei were fixed, obviating the need for Ehrenfest dynamics. When comparative simulations were performed treating the H_2_ nuclei classically using conventional electronic RT-TDDFT, the hydrogen nuclei moved according to Ehrenfest dynamics. The quantum treatment of the hydrogen nuclei results in faster H_2_ photodissociation and larger isotope effects (Figure 8).^168^ This behavior likely stems from a combination of enhanced excited-state electronic coupling between the plasmonic mode and the H_2_ antibonding orbital due to proton delocalization and ZPE effects. In another study, we applied RT-NEO-TDDFT to an HF molecule placed near a one-dimensional Au nanowire composed of up to 34 Au atoms, showing that the infrared plasmonic motion within the Au nanowire can resonantly transfer electronic energy to the HF proton vibrational stretch mode.^169^ These two studies illustrate that the RT-NEO method is well-suited for investigating plasmonic chemistry using atomic-level metallic systems.

**Figure 8:**
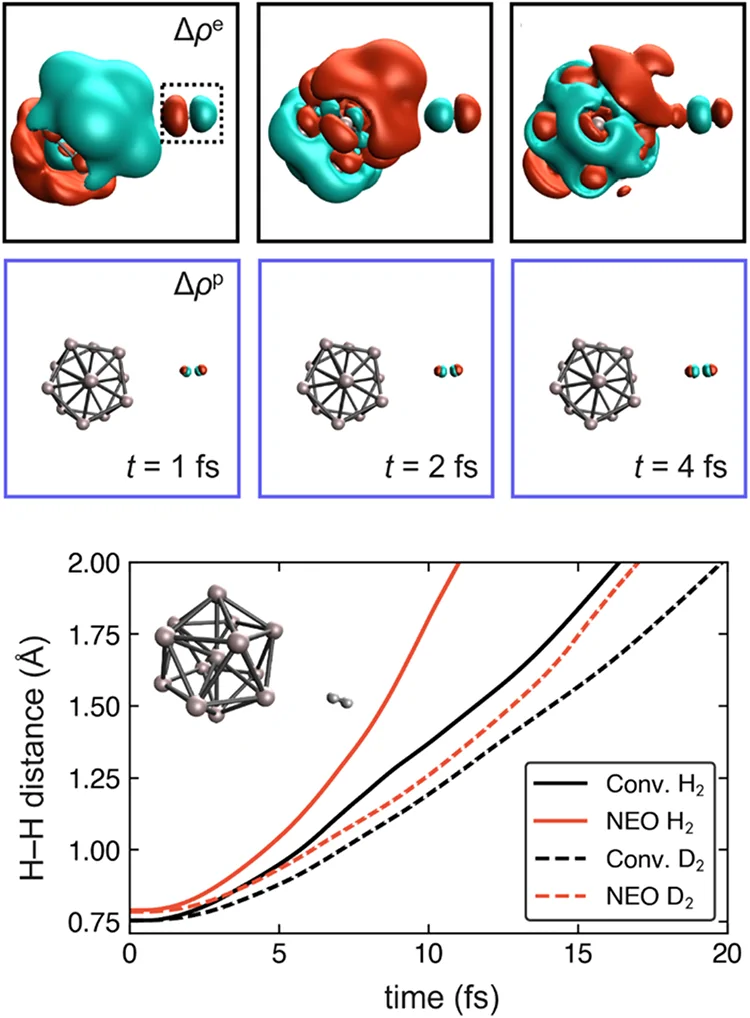
Application of RT-NEO-TDDFT to plasmon-induced H_2_ dissociation near an Al_13_^–^ cluster. The metal nuclei are fixed, and the dynamics of the H_2_ or D_2_ are monitored following illumination by a Gaussian pulse. Top: electronic and protonic density difference plots at the time indicated, where red (cyan) represents the increased (decreased) densities. The large-scale electronic density redistribution in the Al_13_^–^ cluster is a signature of a localized surface plasmonic resonance excitation. Bottom: comparison of dynamics computed using RT-NEO-TDDFT with quantized H_2_ and D_2_ nuclei or using conventional RT-TDDFT calculations with classical H_2_ and D_2_ nuclei. Quantization with the NEO method speeds up photodissociation and increases the kinetic isotope effect. Adapted with permission from ref 168. Copyright 2023 American Chemical Society.

### NEO-TDCI

Although RT-NEO-TDDFT is a powerful and computationally efficient method, it also has serious limitations. The main limitations relevant to our applications are that it cannot describe multireference problems, such as deep hydrogen tunneling, and it cannot describe double excitations, such as double electron–proton vibronic excitations. To address these challenges, we have developed the NEO-TDCI method.^158^ The wave function ansatz for NEO-TDCI is eq ([Disp-formula eq3]), where the expansion coefficients are assumed to be time-dependent. Substituting this wave function into the time-dependent Schrödinger equations leads to the NEO-TDCI equations for the numerical propagation of the time-dependent expansion coefficients.

The main features of NEO-TDCI can be showcased using the model system HeHHe^+^ with the He atoms fixed to a distance associated with hydrogen tunneling.^158^ At this distance, the hydrogen moves in a symmetric double-well potential on the Born–Oppenheimer potential energy surface. The lowest two vibronic states correspond to symmetric and antisymmetric wave functions with bilobal proton densities, similar to those shown for FHF^–^ in Figure 3. NEO-TDCI can be used to describe hydrogen tunneling dynamics in this system. Starting a NEO-TDCI trajectory with the initial wave function corresponding to the proton density localized near one of the He atoms, the proton becomes bilobal and delocalized between both He atoms and then becomes localized near the other He atom and continues to oscillate back and forth between the two He atoms with a frequency directly related to the tunneling splitting energy (Figure 9A).

**Figure 9:**
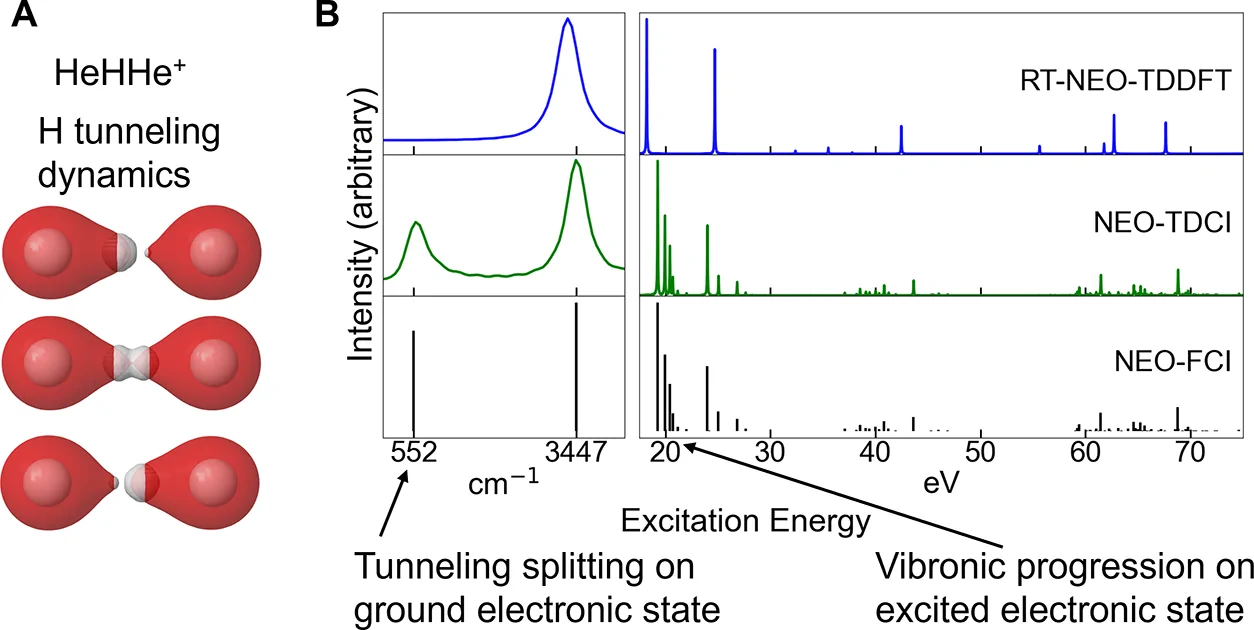
Application of NEO-TDCI to hydrogen tunneling dynamics and vibronic spectroscopy. (A) Hydrogen tunneling dynamics in HeHHe^+^, where the proton density (white) oscillates between the fixed He atoms, becoming delocalized in the middle. (B) Vibronic spectra of HeHHe^+^ showing that NEO-TDCI reproduces the peak associated with the tunneling splitting on the ground electronic state and the vibronic progression associated with double electron–proton excitations to a series of excited proton vibrational states on the excited electronic state. These features agree with NEO-FCI and cannot be described by RT-NEO-TDDFT. Adapted with permission from ref 158. Copyright 2024 American Chemical Society.

NEO-TDCI can also be used to compute vibronic spectra via the Fourier transform of the time-dependent total dipole moment, including both electronic and protonic dipole moment contributions.^158^ For HeHHe^+^, the spectrum can be compared to results from time-independent NEO full configuration interaction (NEO-FCI) calculations, which are exact for the chosen basis sets. For this system, NEO-TDCI describes two distinct features that are present in the NEO-FCI spectrum but are absent from the RT-NEO-TDDFT spectrum (Figure 9B). In the low-energy region, the NEO-TDCI spectrum shows a peak corresponding to the hydrogen tunneling splitting on S_0_, which is the ground electronic state. In the high-energy region, the NEO-TDCI spectrum shows a vibronic progression corresponding to excitations from the ground vibrational state on S_0_ to the ground and excited proton vibrational states on S_1_, which is the excited electronic state. Both features agree quantitatively with the NEO-FCI spectrum and cannot be described by RT-NEO-TDDFT. Additionally, NEO-TDCI can be used to compute time-resolved vibronic spectra.^170^ For this purpose, a NEO-TDCI trajectory can be driven by an applied field that induces excitations, and absorption spectra can be generated with NEO-TDCI from complex-valued time-dependent NEO-CI wave functions extracted along the trajectory. These simulations produce two-dimensional spectra that depend on both time and energy.^170^

A challenge of NEO-TDCI is that the quality of the results depends on selecting physically meaningful configurations for the expansion. In practice, these configurations can be obtained from NEO-CASSCF calculations. To avoid the choice of active spaces, all excitations of certain types could be included (e.g., all single electron and single proton excitations from a NEO-HF reference). Furthermore, in the applications of NEO-TDCI discussed here, the classical nuclei are fixed, which can describe ultrafast processes for which the electronic and protonic densities move in a predominantly fixed field of the other nuclei. For slower processes that entail movement of the classical nuclei, the real-time dynamics can be propagated on adiabatic NEO-CI vibronic surfaces using a nonadiabatic dynamics method, such as Ehrenfest or surface hopping. Thus, NEO-TDCI is a useful tool for simulating both spectroscopy and dynamics of ultrafast processes.

## Polaritonic Chemistry

The NEO framework is particularly well-suited for studying polaritonic chemistry. Molecular polaritons are hybrid light–matter states arising from strong light–matter interactions.^171–173^ In optical cavities, the cavity mode can be tuned to be resonant with either an electronic or a vibrational excitation of a molecule, corresponding to electronic strong coupling (ESC) or vibrational strong coupling (VSC). This strong coupling leads to a splitting of the peak associated with the electronic or vibrational excitation into upper and lower polariton peaks separated by the energy associated with the Rabi frequency. A wide range of theoretical methods have been developed to study polaritons,^173^ but a review of these methods is beyond the scope of this Perspective. The NEO framework enables the first-principles dynamical treatment of ESC and VSC on the same level in a consistent manner. The electrons and specified nuclei are treated quantum mechanically, while the other nuclei are treated classically, and the cavity modes can be treated either classically or quantum mechanically.

Semiclassical NEO dynamics methods for polaritons have been developed using both RT-NEO-TDDFT and NEO-TDCI.^174,175^ In these approaches, the molecular system is described by RT-NEO-TDDFT or NEO-TDCI, adding a term to the time-dependent equations that accounts for the coupling between the molecular dipole moment and the cavity mode. The cavity mode is propagated according to classical equations of motion, including an additional term corresponding to its coupling with the molecular dipole moment. A self-consistent propagation scheme is used to numerically solve the quantum and classical equations numerically. We have shown that these semiclassical RT-NEO methods can produce physically reasonable Rabi splittings for both ESC and VSC (Figure 10A). Beyond polaritonic spectra, RT-NEO methods also enable the study of real-time dynamics in cavities. We performed semiclassical RT-NEO-TDDFT calculations of *o*HBA in a cavity with the cavity mode tuned to the π–π* electronic excitation that induces excited-state intramolecular proton transfer.^174^ Our calculations showed that the cavity mode can suppress photoinduced proton transfer under certain conditions.

**Figure 10:**
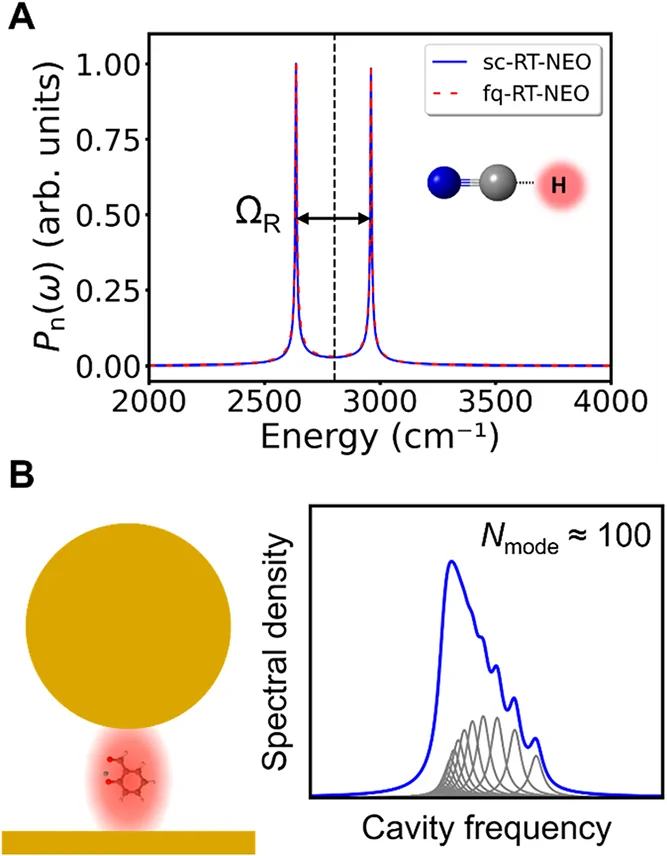
Application of RT-NEO-TDDFT to polaritonic chemistry. (A) Spectrum of HCN in a cavity with the cavity mode tuned to the C–H bending mode under VSC. The dashed line indicates the energy of this vibrational peak outside the cavity, and the splitting of this peak indicates polariton formation, where the upper and lower polariton peaks are separated by the energy associated with the Rabi frequency Ω_R_. The spectrum is the same when the cavity mode is treated classically or quantum mechanically (labeled sc and fq, respectively, for semiclassical and full quantum). However, the full quantum treatment allows light–matter entanglement. (B) Semiclassical RT-NEO-TDDFT study of excited-state proton transfer in a plasmonic nanocavity. Adapted with permission from refs. 176 and 178. Copyright 2025 and 2026 American Chemical Society.

As discussed above, NEO-TDCI enables the study of double electron–proton vibronic excitations and hydrogen tunneling. We performed semiclassical NEO-TDCI calculations with the cavity mode tuned to a vibronic excitation associated with an excited vibrational state on the excited electronic state.^175^ In the vibronic strong coupling regime, we observed off-resonant effects on the vibronic spectrum in addition to the standard Rabi splitting. For a tunneling system, when the cavity mode was tuned to the tunneling splitting, our semiclassical NEO-TDCI calculations showed that the cavity mode can alter the hydrogen tunneling dynamics.^175^

We have also developed RT-NEO methods for polaritonic systems in which the cavity mode is treated quantum mechanically.^176,177^ This method requires the propagation of a joint molecule–mode density matrix and is more computationally expensive than the semiclassical approaches. However, these fully quantum methods allow for molecule–mode quantum entanglement effects in real-time dynamics. Our application of this method to the HCN molecule under VSC showed that the Rabi splitting and polariton peak locations are nearly identical for the classical and quantum treatments of the cavity mode (Figure 10A).^176^ However, the quantum treatment of the cavity mode showed evidence of light–matter entanglement that can be probed via the von Neumann entropy.

In addition to these simulations of an optical cavity with a single mode, we have also developed methods to model a multimode plasmonic nanocavity.^178^ An advantage of plasmonic nanocavities is that they can achieve strong coupling on the molecular scale with either one or a few molecules.^179^ However, plasmonic nanocavities are challenging to model due to their multimodal character and short cavity lifetimes.^180,181^ To address these challenges, we coupled the RT-NEO-TDDFT method to multiple classical cavity modes (e.g., 100 modes) representing a spectral density and added cavity loss to the time-dependent equations. This approach allowed us to model an experimentally relevant gold nanoparticle-on-mirror system using realistic parameters for the spectral density (Figure 10B).^178^ By placing *o*HBA or a related molecule inside the plasmonic nanocavity, we showed that excited-state intramolecular proton transfer dynamics can be probed through cavity emission. Under strong coupling conditions, the cavity can modify the excited-state proton transfer dynamics and, in some cases, suppress proton transfer. All of these different types of polariton calculations highlight the power of RT-NEO methods for studying polaritonic chemistry.

## Remaining Challenges and Future Directions

This Perspective summarizes the main NEO methods available and highlights the wide range of applications that have been explored. For each application, the NEO calculations provide fundamental insights into the underlying physical principles such as the structural role of nuclear delocalization in hydrogen-rich materials under high pressure or the mechanistic role of hydrogen tunneling in chemical reactions. An important aspect of these NEO methods is that they are accessible to nonexperts with currently available software packages. For example, a NEO-DFT calculation is nearly as straightforward to perform as a conventional DFT calculation. The user simply needs to select the quantum nuclei, along with nuclear basis sets and an electron–proton correlation functional. The availability of NEO-DFT, as well as wave function methods such as NEO-CCSD, in popular quantum chemistry software packages may facilitate this approach becoming mainstream.

Despite these successes, many challenges in this field remain. For NEO-DFT, a central challenge is the development of electron–proton correlation functionals. Although the epc17-2 functional provides a qualitatively reasonable description of zero-point energy and nuclear delocalization, it is not quantitatively accurate. Analogous to the development of a plethora of electronic exchange–correlation functionals, substantial effort must be directed toward the development of more accurate and flexible electron–proton correlation functionals. Another major challenge is the development of alternative protonic basis sets, along with revised electronic basis sets that are suitable for NEO calculations. Standard electronic basis sets were developed for point charge nuclei and should be revised to account for delocalized nuclei within the NEO framework.^64^ In principle, electronic and protonic basis sets should be developed simultaneously in a consistent manner. Machine learning methods may be useful for the basis set and functional developments, but these methods require a suitable data set. In principle, the data set should include both ground and excited-state properties, possibly generated with numerically exact grid-based methods, and should be generated using wave function methods to avoid dependence on the choice of density functionals.

The treatment of only specified nuclei quantum mechanically enables the NEO method to be computationally efficient for large systems. However, this distinct treatment of specified nuclei also introduces an inconsistency among the nuclei. The mass ratio between heavy nuclei and hydrogen is much smaller than the mass ratio between a proton and an electron. The non-Born–Oppenheimer effects between the classical and quantum nuclei can be included via nonadiabatic dynamics methods such as Ehrenfest or surface hopping. Ehrenfest dynamics is well-suited for spectroscopy simulations and certain types of chemical processes but cannot describe branching processes effectively, whereas surface hopping accurately describes branching processes but is less useful for spectroscopy simulations. Thus, the appropriate method must be chosen for a given application. Another option is to combine NEO with instanton theory or other types of path integral methods. The NEO formalism can also treat multiple types of nuclei, such as hydrogen and deuterium, as well as positrons and muons, quantum mechanically within the same system. Alternatively, it is possible to treat *all* nuclei quantum mechanically within the NEO framework if certain approximations are made.^35^ The quantum treatment of all nuclei may be computationally tractable, but the basis sets and correlation must be treated carefully to ensure sufficient accuracy.

Although NEO-DFT is the workhorse for large-scale applications in chemistry, biology, and materials, NEO wave function methods must be further developed to enable applications to larger, more relevant systems. NEO-CCSD has been applied to relatively large water clusters, but NEO-MRCI and NEO-TDCI applications have been limited to relatively small systems such as triatomic molecules and malonaldehyde. Quantum embedding and orbital localization methods^46^ may facilitate the extension of these NEO wave function methods to larger systems. Another promising direction is quantum computing, which has been explored within the NEO framework in several initial studies.^182–184^ Lastly, the investigation of magnetic field and spin effects is an exciting direction. A preliminary study of magnetic field-driven real-time quantum dynamics within the NEO framework^185^ lays the groundwork for future studies of molecules in magnetic fields, with implications for NMR and magnetic vibrational circular dichroism. The field of multicomponent quantum chemistry is maturing, but many new directions are still emerging, providing ample opportunities for creativity and innovation.
